# Simple and efficient protocol to isolate and culture brain microvascular endothelial cells from newborn mice

**DOI:** 10.3389/fncel.2022.949412

**Published:** 2022-10-13

**Authors:** Priscila Nicolicht-Amorim, Lina M. Delgado-Garcia, Thabatta Karollynne Estevam Nakamura, Natália Rodrigues Courbassier, Amanda Cristina Mosini, Marimelia A. Porcionatto

**Affiliations:** ^1^Laboratory of Molecular Neurobiology, Department of Biochemistry, Universidade Federal de São Paulo, São Paulo, Brazil; ^2^Laboratory of Neurobiology, Department of Physiology, Universidade Federal de São Paulo, São Paulo, Brazil

**Keywords:** cerebrovascular endothelial cells, primary culture protocol, non-neuronal cells isolation, *in vitro* blood-brain barrier, neurovascular unit

## Abstract

The neurovascular unit (NVU) is a multicellular structure comprising of neurons, glial cells, and non-neural cells, and it is supported by a specialized extracellular matrix, the basal lamina. Astrocytes, brain microvascular endothelial cells (BMECs), pericytes, and smooth muscle cells constitute the blood–brain barrier (BBB). BMECs have a mesodermal origin and invade the nervous system early in neural tube development, forming the BBB anatomical core. BMECs are connected by adherent junction complexes composed of integral membrane and cytoplasmic proteins. *In vivo* and *in vitro* studies have shown that, given the proximity and relationship with neural cells, BMECs acquire a unique gene expression profile, proteome, and specific mechanical and physical properties compared to endothelial cells from the general vasculature. BMECs are fundamental in maintaining brain homeostasis by regulating transcellular and paracellular transport of fluids, molecules, and cells. Therefore, it is essential to gain in-depth knowledge of the dynamic cellular structure of the cells in the NVU and their interactions with health and disease. Here we describe a significantly improved and simplified protocol using C57BL/6 newborn mice at postnatal day 1 (PND1) to isolate, purify, and culture BMECs monolayers in two different substrates (glass coverslips and transwell culture inserts). *In vitro* characterization and validation of the BMEC primary culture monolayers seeded on glass or insert included light microscopy, immunolabeling, and gene expression profile. Transendothelial electrical resistance (TEER) measurement and diffusion test were used as functional assays for adherent junction complexes and integrity and permeability of BMECs monolayers. The protocol presented here for the isolation and culture of BMECs is more straightforward than previously published protocols and yields a high number of purified cells. Finally, we tested BMECs function using the oxygen–glucose deprivation (OGD) model of hypoxia. This protocol may be suitable as a bioscaffold for secondary cell seeding allowing the study and better understanding of the NVU.

## Introduction

Since the establishment of the concept of the Neurovascular Unit (NVU) by the Stroke Progress Review Group^1^ (National Institute of Health, 2001^2^), the relationship between neural networks and vasculature has gained increased attention. The NVU is a multicellular and dynamic structure comprised of neural (neurons, astrocytes, and oligodendrocytes) and non-neural cells (mainly brain microvascular endothelial cells or BMECs, pericytes, smooth muscle cells, and microglia), supported by the basal lamina, a specialized extracellular matrix (Abbott et al., 2010; Muoio et al., 2014; Iadecola, 2017; Sweeney et al., 2019; Schaeffer and Iadecola, 2021; Soto-Rojas et al., 2021; Dong et al., 2022). The primary functions of the NVU include modulating vascular permeability, cerebral hyperemia, and immune response, which are essential for maintaining central nervous system function (Blanchette and Daneman, 2015; Liebner et al., 2018; Soto-Rojas et al., 2021; Dong et al., 2022). Cerebral hyperemia allows the supply of oxygen and glucose to the nervous tissue, and, under physiological conditions, the human brain consumes about 20% of the total body circulating oxygen (Muoio et al., 2014; Iadecola, 2017; Schaeffer and Iadecola, 2021).

BMECs play a fundamental role in both, immature and mature nervous systems. BMECs have a mesodermal origin and invade the nervous system in the early stages of neural tube development, forming the blood–brain barrier's anatomical core (BBB). During these initial stages, BMECs display a non-specialized phenotype and lower gene expression of constitutive proteins, which may compromise BBB's integrity and permeability, exposing the brain to xenobiotics and making it vulnerable to inflammation (Strazielle and Ghersi-Egea, 2015; Sweeney et al., 2018; Ahmad et al., 2020; Yu et al., 2020; Eng et al., 2022; Lye et al., 2022). However, after the remodeling and maturation of brain regions, BMECs specialize and adapt to diverse phenotypes, such as veins, arteries, and capillaries (Hogan et al., 2004; Fantin et al., 2013; Engelhardt and Liebner, 2014; Dejana et al., 2017). Additionally, interaction with diverse neural cell types and cell–cell chemical signaling exchange impact their gene expression profile, proteome, and mechanical and physical properties (Abbott, 2002).

BMECs build the blood vessel wall, regulate molecule movements, and transport, and produce extracellular matrix compounds (Cai et al., 2017; Iadecola, 2017; Bell et al., 2020). BMECs are connected by complexes of integral membrane and cytoplasmic proteins. Specifically, tight junction proteins prevent the paracellular transport of molecules and seal the clefts, while adherent junctions perform cell-cell adhesion and promote cell maturation (Liebner et al., 2018; Langen et al., 2019; Profaci et al., 2020). In addition, transporters regulate intracellular influx and efflux of specific substrates and limit the entry of xenobiotics and endogenous molecules (Hindle et al., 2017; Sweeney et al., 2019). BMECs promote low permeability and high transendothelial resistance (Blanchette and Daneman, 2015; Daneman and Prat, 2015; Liebner et al., 2018; Profaci et al., 2020; Takata et al., 2021) in BBB and are fundamental in maintaining brain homeostasis by regulating transcellular and paracellular transport of fluids, molecules, and cells. However, BMECs malfunction may cause the progressive loss and degeneration of the NVU, a condition observed in many neurological disorders, such as stroke, brain injury, cancer, and neurodegenerative diseases such as Alzheimer's disease (De Luca et al., 2020; Schaeffer and Iadecola, 2021; Soto-Rojas et al., 2021). Xenobiotics can cause deleterious changes in brain maturation that can lead to the development of neurological disorders throughout life (Strazielle and Ghersi-Egea, 2015; Eng et al., 2022; Lye et al., 2022). Adult BMECs provide essential neuroprotection against environmental toxins through elevated levels of efflux transporters (P-gp/ABCB1, MRP1/ABCC1, BCRP/ABCG2) (Gomez-Zepeda et al., 2019). Moreover, in the face of neurological disorders, transporters tend to increase their expression, leading to great difficulty in testing new therapeutic drugs (Strazielle and Ghersi-Egea, 2015; Gil-Martins et al., 2020). Late fetal and postnatal BMECs show low expression levels of Glycoprotein-P (P-gp), one of the ABC transporters subunits, which may result in increased permeability of the BBB exposed to xenobiotics, stress, and inflammatory stimuli (Goralski et al., 2006; Gil-Martins et al., 2020; Eng et al., 2022). Most of the BBB *in vitro* models available are BMECs obtained from adult animals, leading us to identify the need for developing a model that would mimic the newborn BBB. Thus, this study aimed to standardize a protocol to isolate and culture BMECs obtained from neonatal mice at postnatal day 1 (PND1) to assemble a 2D BBB. Published protocols require a large number of animals, expensive material, and substantial cell manipulation (Aspelund et al., 2015; Daneman and Prat, 2015; Louveau et al., 2015; Zhao et al., 2015; Stamatovic et al., 2016; Jiang et al., 2018; Wong et al., 2019). Here, we describe a significantly improved, minimally manipulative, and simplified protocol to isolate, purify, and culture BMECs from newborn mice.

*Application*. This protocol can be used jointly with various techniques, from cell co-culturing and cell imaging to molecular biology, biochemistry, pharmacology, genetics, electrophysiological studies, and *in vivo* transplantation assays. *Advantages*. We describe a simple and efficient protocol for BMECs primary culture from newborn mice (PND1). Comparative analyses revealed that the BMECs isolation and culture protocols described here yield more purified cells than the published protocol used as a reference (Xue et al., 2013). The protocol described here is suitable to be performed using newborn mice, while most protocols use adult mice or rats. In addition, it reduces the number of animals needed and requires minimally manipulative procedures. BMECs culture system efficiency was determined by immunocytochemistry, gene expression, TEER, and permeability analyses were equivalent to other existent protocols. *Limitations*. The major limitation faced in this and other protocols of BMECs primary culture is the contamination with astrocytes and other neural and non-neural cells.

## Methods

### Animal research ethics

This protocol was approved by the Ethics Committee on the Use of Animals of the *Universidade Federal de São Paulo* (CEUA/1344290719). We used C57BL/6 newborn mice at PND1 obtained from *Centro de Desenvolvimento de Modelos de Experimentação (CEDEME), Escola Paulista de Medicina, Universidade Federal de São Paulo*.

### Step-by-step protocol for BMECs primary culture

**Figure 1:**
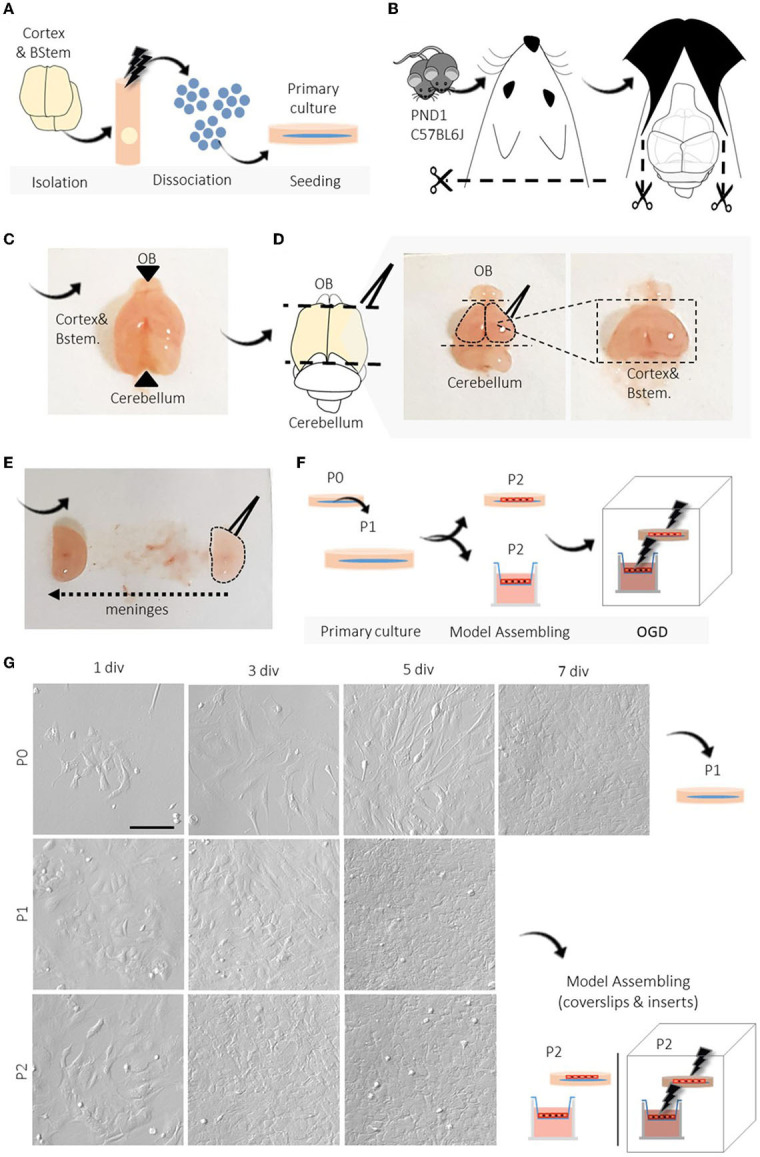
Overview of BMECs protocol from newborn mice and primary culture. **(A–F)** Schematic representation of the primary step-by-step procedures. **(G)** Representative light microscopy images of the progression and evaluation of the cell culture after isolation, P0, P1, and P2. During each passage (P0, P1, P2), we photographed the cell cultures at different periods until reaching confluence (1, 3, 5, and 7 div). N =3. PND1, postnatal day 1; OGD, oxygen-glucose deprivation; div, days in vitro; P, passage; SD, standard deviation. Scale bar 100 μm.

The suggested amounts of reagents are suitable for the isolation, dissociation, and seeding of BMECs obtained from two C57BL6J mice (PND1) cortices and yield approximately a total of 1.2–1.5 × 10^7^cells/ml at passage two P2 (Figures 1A–F). The equipment, reagents, and recipes used are described in Tables 1–3.

1. *Brain isolation and tissue dissection*. Decapitate the mice and dissect the brains, under sterile conditions. Then, separate the olfactory bulbs and cerebellum with a spatula and tweezers. Next, remove the meninges by rolling the brain across a sterile filter paper (Figures 1B–E).
2. Store both tissue samples in a microtube containing 2 ml of cold Hank's balanced salt solution (HBSS) with 1% penicillin/streptomycin (P/S) solution (8–15°C).
3. *Dissociation*. Under the culture hood, shred the tissue with sterile tweezers for mechanical dissociation. After decanting the tissue, carefully remove the solution and add 1 ml of HBSS to wash the tissue. Homogenize and transfer to a 15 ml conical tube. Let stand until the pellet is decanted, then remove the supernatant.
4. For the first round of enzymatic dissociation, add 1 ml of 10 × Trypsin/Versene solution (1:1) and incubate at 37°C for 20 min. *Critical step:* Make sure the solution covers the tissue and carefully homogenize the tube by inverting it every 5 min. After incubation, the tissue should have a cloud-like appearance.
5. After incubation, add 1 ml of fetal bovine serum (FBS) to block trypsin activity. Then, homogenize and mechanically dissociate the tissue by pipetting the cells up and down using p 1000 and p 200 tips sequentially. *Critical step:* Be careful not to produce bubbles.
6. Centrifuge 800 × g for 5 min at room temperature (20–25°C) and discard the supernatant. *Critical step:* Be careful not to remove the suspended myelin, which contains the vascular tissue. Add 2 ml of 25% bovine serum albumin (BSA) and carefully homogenize the solution.
7. Centrifuge 1,500 × g for 15 min at room temperature (20–25°C) and discard the supernatant. *Critical step:* Be careful not to remove the supernatant myelin, as the interest is the recovery of the vessels.
8. For the second round of enzymatic dissociation and myelin separation, add 1 ml of Type IV Collagenase/Versene solution and incubate at 37°C for 30 min.
9. Add 1 ml of FBS to block the collagenase activity. Homogenize and clean the solution by filtering through a 10 nm sterile mesh adapted to a 50 ml conical tube. Wash the mesh with an additional 1 ml of FBS or medium to recover more cells. Finally, centrifuge 800 × g for 15 min at room temperature and discard the supernatant.
10. *Maintenance of the primary cell culture and BMECs enrichment*. Suspend the pellet in 5 ml of Dulbecco's Modified Eagle Medium (DMEM)/F-12 with 20% FBS, 2% L-glutamine, 1% P/S solution supplemented with 1.0 ng/ml of human basic fibroblast growth factor (bFGF) and 20.0 μg/ml of bovine sodium heparin, and seed in a T25 culture flask previously treated with 1% gelatin. Change the medium the next day, and then every 2–3 days. Incubate cells at 37°C, 95% humidity, and 5% CO_2_.
11. BMECs enrichment is obtained through passaging. For this purpose, perform the first passage within 5–7 days of culture to prevent astrocytes and pericytes growth. Add 2 ml of 10 × Trypsin/Versene solution (1:1) and incubate at 37°C for 6 min. *Critical step:* Ensure the solution is in contact with the entire culture flask surface. After incubation, you should note that the cells are detached. You may want to verify the effectivity of the incubation under a light microscope.
12. After incubation, add 1 ml of FBS to block trypsin activity. Homogenize and transport the solution to a 15 ml conical tube. Centrifuge at room temperature at 400 × g for 5 min and discard the supernatant.
13. Suspend the pellet in 5 ml of DMEM/F12 with 20% FBS, 2% L-glutamine, 1% P/S solution supplemented with 20 ng/ml bFGF, and 20.0 μg/ml bovine sodium heparin and split the solution between two T25 culture flasks previously treated with 1% gelatin. The addition of bFGF contributes to the purification of BMEC primary culture. Incubate cells at 37°C, 95% humidity, and 5% CO_2_ for 5–6 days until the following passage (P2).
14. *Model assembling on glass coverslips and transwell culture inserts*. For BMECs culture in coverslips, inserts, and 96-well plates, follow steps 11 and 12 and incubate at 37°C, 95% humidity, and 5% CO_2_. Evaluate the progression of the culture by light microscopy. Check cell viability, growth rate, and purity. For 13 mm-coverslips (pre-treated with 1% gelatin), count and seed 2 × 10^5^ cells in 500 μl of the medium. For BMECs culture in polycarbonate inserts (0.47 cm^2^ in surface area, 0.4 μm pore, pre-treated with poly-L-lysine), count and seed 2 × 10^5^ cells in 700 μl of the medium. We suggest seeding the cells on the insert's apical side and incubating them for 24 h. *Critical step*. The polycarbonate inserts do not allow a proper image by light microscopy. Therefore, you may want to supervise the progress of the culture by seeding BMECs from the same sample in one or two coverslips. Coverslips culture may give you an idea of the cell viability, growth rate, and purity of the BMECs in the insert. For cell viability in 96-well plates pre-treated with 1% gelatin, count and seed 1.5 × 10^4^ cells/cm^2^ in 100 μl of the medium.

**Table 1:** Equipment's.

**Equipment**	**Manufacturer**	**Catalog number**
7500 Fast Real-Time PCR System	ThermoFisher	4357362
Centrifuge	Eppendorf	5702
DNM-9602 Microplate Reader Spectrophotometer	Perlong	M10814070202
Forma^TM^ Series 3 Water Jacketed CO_2_ Incubator	ThermoFisher	4110
Hypoxia Incubator Chamber	STEMCELL	27310
Inverted Microscopes	Olympus	IX2-ILL100
Microplate Reader	Greiner Bio-One	655209
Millicel ^®^ ERS-2 Voltmeter	MERCK	MERS00002
NanoDrop^TM^ One/One^C^ Spectrophotometer	ThermoFisher	ND-ONE-W
SimpliAmp^TM^ Thermal Cucler	ThermoFisher	A24812
Single Flow Meter	STEMCELL	27311
SpectraMax^®^ M3 Spectrophotometer	Molecular Devices	MT 05123
Zeiss Microscopy LSM 780	Carl Zeiss GmbH	N/A

**Table 2:** Reagents.

**Reagents**	**Manufacturer**	**Catalog number**
Alexa Fluor^TM^ 488—Goat anti-Rabbit	ThermoFisher	A11008
Alexa Fluor^TM^ 594—Donkey anti-Mouse	ThermoFisher	A21203
Alexa Fluor^TM^ 647—Goat anti-Chicken	ThermoFisher	A21449
Bovine Serum Albumin (BSA)	Sigma Aldrich	A3294-100G
Bovine Sodium Heparin	Kin Master	50.1000.01
CellTiter 96^®^ AQueous One Solution Reagent kit	Promega	G35582
Collagenase, Type IV	ThermoFisher	17104019
DAPI	Sigma Aldrich	D9542
DMEM/F12	ThermoFisher	12500-062
Fast SYBR^TM^ Green Master Mix	ThermoFisher	4385617
Fetal Bovine Serum (FBS)	ThermoFisher	12657029
FITC-DEXTRAN 4 KDa	Sigma Aldrich	46944
Fluoromount-G^TM^ Mounting Medium	ThermoFisher	00-4958-02
Gelatin from porcine skin	Sigma Aldrich	G2500
High-Capacity cDNA Reverse Transcription Kit	ThermoFisher	4374966
Human FGF-basic	ThermoFisher	AA 10-155
L-glutamine	MP Biomedicals	101806
Normal Goat Serum	ThermoFisher	PCN5000
Nunc^TM^ Polycarbonate Cell Culture Inserts	ThermoFisher	140620
Oxoid^TM^ Resazurin Anaerobic Indicator	ThermoFisher	BR0055B
Penicillin/streptomycin	ThermoFisher	15140122
Primary—GFAP antibody	Merck Millipore	AB5541
Primary—NG2 antibody	Merck Millipore	AB5320
Primary—ZO-1 Monoclonal antibody	ThermoFisher	33-9100
Poly-L-Lysine	Sigma Aldrich	P2636
PureLink^TM^ RNA Mini kit	ThermoFisher	12183018A
SILAC Advanced DMEM/F-12 Flex Media, no glucose, no Phenol red	ThermoFisher	A2494301
Triton^TM^ X-100	Sigma Aldrich	9002-93-1
Trypsin (2.5%)	ThermoFisher	15090046

**Table 3:** Recipes.

**Recipes**	**Final concentration**
Hank's balanced salt solution (HBSS)	5.36 mM KCl
	0.44 mM KH_2_PO_4_
	4.16 mM NaHCO_3_
	136.9 mM NaCl
	0.336 mM Na_2_PO_4_
	5.55 mM glucose
	Ca^+2^ and Mg^+2^ free
Versene	2.7 mM KCl
	1.8 mM KH_2_PO_4_
	136.9 mM NaCl
	10.0 mM Na_2_HPO_4_
	0.68 mM EDTA

### Validation analysis and functional assay: Integrity and permeability assessment of BMECs monolayers in inserts

#### Transendothelial electrical resistance

The Transendothelial Electrical Resistance (TEER) measurement was used to evaluate the integrity of the monolayer formed by the BMECs in the inserts. The measurements of the BMECs monolayers were recorded on days 2, 3, 5, 10, and before/after 4-h OGD using a pair of STX2 electrodes connected to a Millicell ERS-2 Voltmeter System. We registered at least three times the same insert and calculated the mean value (*BMECs insert value*). Additionally, we recorded the control values using insert without cells (*cell-free insert value*). Finally, we normalized the reads, calculating the difference between *cell-free insert values* from *BMECs insert values* multiplied by the insert area (0.47 cm^2^).


$$\begin{array}{l} TEER\;(\Omega \;\times cm^{2})=(\;BMECs\;insert\;value-\;cell\;free\;insert\\ \;value)\times \;area\;\left(cm^{2}\right) \end{array}$$

Due to the small number of studies evaluating TEER measurements in BMECs from newborn mice (PND1), we used a human umbilical vein endothelial cell (HUVEC) line culture to validate our methodology (Supplementary Figure 3). In addition, we recommend adapting the electrodes in an arm-holding structure to facilitate positioning and stabilization of the measurements (Supplementary Figure 4). In the case of TEER measurements for the OGD hypoxia model, we considered two experimental designs: a paired sampling, named before and after OGD, and alternative, and independent sampling, named control and OGD. FITC-Dextran permeability test is taken after TEER measurement in control and OGD samples.

#### Diffusion (volume retention) and FITC-DEXTRAN assays

We used a 24-h diffusion test and 2-h fluorescein isothiocyanate-dextran (FITC-Dextran, MW = 4 kDa) permeation experiment to analyze the paracellular permeability of the barrier of BMECs. For the diffusion assay, BMECs were seeded at a density of 2 × 10^5^ cells/cm^2^ on the insert apical side grown in 24-well plates. We adapted the diffusion test from Xue et al. (2013) and Wang et al. (2015) protocols to measure the diffusion rate between the apical and basolateral insert. After 3 and 10 days of culture, 700 μl of DMEM/F12 were added to the apical side of the insert containing cells and three inserts without cells as a control. The volume that passed through the inserts after 24-h incubation at 37°C under standard culture conditions was carefully recovered and registered. Additionally, the values from control free-cell inserts (*n* = 3) were registered and used to normalize the results. Furthermore, we performed FITC-Dextran in control (*n* = 6) and OGD groups (*n* = 6) according to the manufacturer's instructions and an adapted protocol from Puscas et al. (2019) and Shan et al. (2019). Briefly, the culture medium in the insert apical side was replaced with a medium supplemented with 100 μg/ml Fluorescein Isothiocyanate FITC-Dextran and incubated for 2 h under normal conditions. After, a 100 μl medium was collected from the basolateral compartment and transferred to a 96-well black plate in triplicate for fluorescence measurement using a microplate reader with an excitation/emission wavelength of 485/520 nm. To calculate FITC-Dextran Permeability (μg/cm^2^), we calculated the ratio of FITC-Dextran collected in the basolateral compartment—FITC-Dextran that passed through the insert—to the surface area of the insert (S, cm^2^). In addition, the permeability coefficient was normalized to the controls to minimize inter-assay variability.


$$Permeability\;=FITC_{basolateral}\;/\;S_{insert}(cm^{2})$$

#### Cell viability analysis—MTS

A total of 1.5 × 10^4^ cells/cm^2^ were plated in quadruplicate 96-well plates for cell viability assessment. Cell viability was assessed using CellTiter 96^®^ AQueous One Solution Reagent kit. The analysis was performed according to the manufacturer's instructions. Briefly, the culture medium of control (*n* = 3) and OGD groups (*n* = 3) at 3 and 10 div was supplemented with 20 μl of CellTiter 96^®^ AQueous One Solution Reagent kit and incubated for 4 h under control and OGD conditions. An additional cell-free well with the medium was used to normalize the values. The viable cells reduce MTS tetrazolium to formazan, a product quantified by measuring the absorbance at 492 nm in a DNM-9602 Microplate Reader Spectrophotometer. Cell viability for both conditions (control and OGD) was calculated by the difference between the obtained absorbance (Abs) and the mean absorbance of the cell-free well. The values were presented as the percentage of cell viability:


$$\begin{array}{l} \%\;Cell\;Viability=(Abs_{control\;or\;OGD\;condition}\\ \;-Mean\;Abs_{cell-free\;well})\;\times 100 \end{array}$$

#### Immunofluorescence and image analysis

Immunofluorescence was used to evaluate BMECs culture to the presence of astrocytes and pericytes. BMECs in both, coverslips and inserts were fixed with 4% paraformaldehyde (PFA) solution for 20 min for immunofluorescence assays. Next, cells were permeabilized with 0.1% Triton X-100 for 5 min. The membrane was cut off the structure to perform immunofluorescence assays of BMECs plated on inserts. After sequential washes with PBS 1 × , the blockage of non-specific sites was performed by incubating with 5% Normal Goat Serum (NGS) for 1 h at room temperature. In sequence, BMECs were incubated with primary antibodies: Glial fibrillary acid protein (GFAP, astrocyte marker, 1:500), Chondroitin Sulfate Proteoglycan (NG2, pericyte marker, 1:200), and Intercellular Junction Protein (ZO-1, BMECs marker, 1:200) diluted in blocking solution incubated overnight, in a humid chamber, at 4°C. Then, cells were washed with PBS 1 × and incubated at room temperature for 1 h with the corresponding secondary antibodies (1:500): Alexa Fluor 488-conjugated goat anti-rabbit IgG, Alexa Fluor 594-conjugated goat anti-mouse IgG, Alexa Fluor 647-conjugated goat anti-chicken IgG and fluorescence nuclear counterstain 4,6-diamidino-2-phenylindole dihydrochloride (DAPI, 1:5,000). Coverslips and inserts were mounted onto slides with Fluoromount G solution. The immunofluorescence was analyzed by confocal microscopy using Zeiss LSM 780 microscope and processed with ImageJ software (1.49v^3^). Quantifying of cell abundance was performed by analyzing the intensity (gray value) for each marker.

#### Quantitative PCR analysis

We evaluated the mRNA expression profile of genes related to tight junctions (tight junction protein 1 and occludin), efflux and glucose transporter (SLC2A1 and ABCB1a), and angiogenesis-hypoxia response (HIF-1α, MMP-2) in control and OGD BMECs. Total RNA extraction was performed using PureLink Mini Kit according to the manufacturer's instructions. The total RNA was quantified, and its quality was assessed with the spectrophotometer NanoDrop One/One system. Ratio readings between 260/280 and 260/230 yield a purity ratio of 1.8–2.0. Then, complementary DNA was synthesized using the High-Capacity cDNA Reverse Transcription Kit. Quantitative PCR was performed using Fast SYBR Green Master Mix in an Applied Biosystems 7500 Real-Time PCR System. Thermal cycling conditions were 95°C for 20 s, 40 × 95°C for 3 s and 60°C for 30 s. The melting curve was performed at 95°C for 1 min, 60°C for 30 s and 95°C for 30 s. The primer sequences are presented in Table 4. The reference gene GAPDH was used to normalize gene expression. We analyzed three biological replicates for each condition and three technical replicates for each gene. ΔCT was used for relative quantification analysis (Livak and Schmittgen, 2001; Schmittgen and Livak, 2008).

**Table 4:** Primers sequence information.

**Gene**	**Reference**	**Protein**	**Function**	**Forward (5^′^-3^′^)**	**Tm (°C)**	**Reverse (3^′^-5^′^)**	**Tm (°C)**	**Size (bp)**
OCLN	NM_008756.2	Occludin	Tight junction	TGAAAGTCCACCTCCTTACAGA	60.1	CGGATAAAAAGAGTACGCTGG	60.7	128
TJP1	NM_009386.2	Tight Junction Protein 1-TJP1	Tight junction	GAGCGGGCTACCTTACTGAAC	62.2	GTCATCTCTTTCCGAGGCATTAG	60.5	75
SLC2A1	NM_011400.3	Glucose transporter protein type 1 GLUT1	Uptake transporter	CTCTGTCGGCCTCTTTGTTAAT	60.0	CCAGTTTGGAGAAGCCCATAAG	60.6	104
ABCB1A	NM_011076.3	ATP Binding Cassette Subfamily B Member 1-(ABC) transporters	Efflux transporter	CTCTATTGGACAAGTGCTCACTG	60.6	CTCCTCGTGCATTGGCGAA	62.7	104
HIF1A	NM_010431	Hypoxia-inducible factor 1-alpha	Hypoxia response	GGGGAGGACGATGAACATCAA	61.5	GGGTGGTTTCTTGTACCCACA	61.9	115
MMP2	NM_008610.3	Gelatinase A Type IV Collagenase	Hypoxia response	CCTGGACCCTGAAACCGTG	62.3	TCCCCATCATGGATTCGAGAA	60.4	96
GAPDH	NM_001289726	Glyceraldehyde-3-phosphate dehydrogenase	Reference Gene	AGGTCGGTGTGAACGGATTTG	63.4	TGTAGACCATGTAGTTGAGGTCA	61.5	123

#### Oxygen-glucose deprivation model of hypoxia

Oxygen-glucose deprivation (OGD) was adapted from Hind et al. (2015). Briefly, the culture medium was replaced with SILAC glucose-free medium and both coverslips and inserts were transferred into a Hypoxia Incubator Chamber. The income valve was connected to a nitrogen tank for oxygen deprivation and adjusted to a flow rate of 25 L/min for 4 min using the Single Flow Meter. After the period, the hypoxia chamber was placed in the incubator at 37°C for 4 h. The hypoxia environment was verified with an anaerobic indicator strip containing resazurin solution. The strip is a color-anerobic indicator, so a color change from pink (high oxygen) to white (low oxygen) is expected after hypoxia. Control cell culture systems were maintained under 37°C, with 95% humidity, and 5% CO_2_.

### Statistical analysis

Statistical analysis and graphical representations were performed using GraphPad Prism (v.5.0, https://www.graphpad.com/scientific-software/prism/). Graphs are presented as mean ± standard error. The difference between groups was assessed using an unpaired Student's t-test and one-way ANOVA. The results were reported in absolute and relative values, and the level of statistical significance adopted was 5% (*p* < 0.05).

## Results and discussion

BMECs form the BBB anatomical core, and the BBB function is to protect the central nervous system (CNS) and depends on cell–cell BMEC interactions mainly of complex tight junction proteins that allow them to maintain a physical barrier. Additionally, efflux and influx transporters maintain CNS homeostasis. Currently, comparable protocols we reviewed require a larger number of animals, more expensive material, and a substantial increase in cell manipulation. Here we described a significantly improved, minimally manipulative, and simplified protocol using newborn mice (PND1) to isolate, purify, and culture BMECs in two different substrates.

### *In vitro* characterization and validation of the primary culture of BMECs monolayers seeded in plastic and insert

Using this protocol, we obtained 1.4 × 10^7^ cells from two PND1 mice brains after isolation, primary culture, and sequential passaging (Tables 5, 6). We developed the protocol using newborn mice because of the specific conditions of the BMECs at early postnatal stages, such as non-specialized phenotype and lower expression of constitutive proteins. We did not test this protocol in adult mice nor used adult mice for BMECs isolation. In addition, the literature is unclear about the total number of cells yielded at specific subculture periods. However, compared to protocols that use adult mice, we used fewer animals per procedure and obtained more cells up to P2 (Tables 5, 6).

**Table 5:** Protocols.

**References**	**Sample**		**Isolation and dissociation**				**Purification and primary culture**			
	**Species; number; Age**	**Tissue**	**Digestion step and** **solutions**	**Blocking** **Solution**	**Manual density-gradient** **separation?**	**Centrifugation** **steps**	**Puromycin purification** **treatment?**	**Confluence (days) and percentag?**	**Experiments and** **assays performed at**	**Density (cells/cm^2^) OR total** **number of cells seeded**
Nakagawa et al. (2009)	Rat; Not informed; 3 weeks	Cortices	Two steps: Collagenase Type II, incubation for 1.5 h; Collagenase dispase, incubation for 1 h	20% BSA	Yes 33% percoll	1,000 × g for 20 min	Yes 4 mg/ml	4; (80%)	Passage 1	1.5 × 10^5^; not informed
Xue et al. (2013)	Rat; Not informed; 2–3 weeks	Brain	Two steps:	10% SFB	Yes	150 × g for 3 min	No	6;	Passage 1	1.0 × 10^5^;
			Trypsin,		10 mm filter	150 × g for 5 min		(80%)		not informed
			incubation for 1.5 h;			600 × g for 15 min				
			Collagenase Type II, incubation for 1 h			150 × g for 5 min				
Burkhart et al. (2015)	Rat; 9–12; 2–3 weeks	Cortices	Two steps: Collagenase Type II, incubation for 75 min; Collagenase dispase, incubation for 50 min	20% BSA	Yes 33% percoll	1,000 × g for 8 min 1,000 × g for 20 min	Yes 4 mg/ml	3; (80%)	Passage 1 (until reaching confluence)	1.0 × 10^5^; P1: 5–8 × 10^6^
Wang et al. (2015)	Rat; 10; 1–3 days	Brain	Two steps: Collagenase type II, incubation for 15 min; Collagenase, incubation for 10 min	20% BSA	No	140 × g for 10 min 1,000 × g for 20 min 140 × g for 10 min	No	7–14; Not informed	Passage 1 (until reaching confluence)	1.0 × 10^7^; Not informed
Bernard-Patrzynski et al. (2019)	Rat; 20; 6 to 8 weeks	Brain	Two steps: Collagenase Type II, incubation for 75 min; Collagenase dispase, incubation for 1 h	20% BSA	Yes 33% percoll	800 × g for 8 min 800 × g for 8 min 1,000 × g for 20 min 30,000 × g for 60 min 1,000 × g for 10 min 800 × g for 8 min	Yes 4 mg/ml	7; (90–95%)	Passage 1, 2, 3	4.5 × 10^5^; Not informed
This work	Mice; 2; 1 day	Brain	Two steps: Trypsin, incubation for 20 min; Collagenase Type IV, incubation for 30 min	20% BSA	Yes 10 mm filter But not necessary	800 × g for 5 min 1,500 × g for 15 min 800 × g for 15 min	No	5–6; (90–95%)	Passage 1, 2	2.0 × 10^5^; P1: 1.1 × 10^7^ (±3.3 × 10^6^); P2: 1.4 × 10^7^ (±6.1 × 10^6^)

**Table 6:** Number of cells.

**Number of cells**	
**Div**	**Mean (SD)**
Isolation (from 2 newborn mice)	4.20 × 10^6^ (±1.5 × 10^6^)
P0	12.8 × 10^6^ (±5.5 × 10^6^)
P1	11.3 × 10^6^ (±3.3 × 10^6^)
P2	14.1 × 10^6^ (±6.1 × 10^6^)

**Figure 2:**
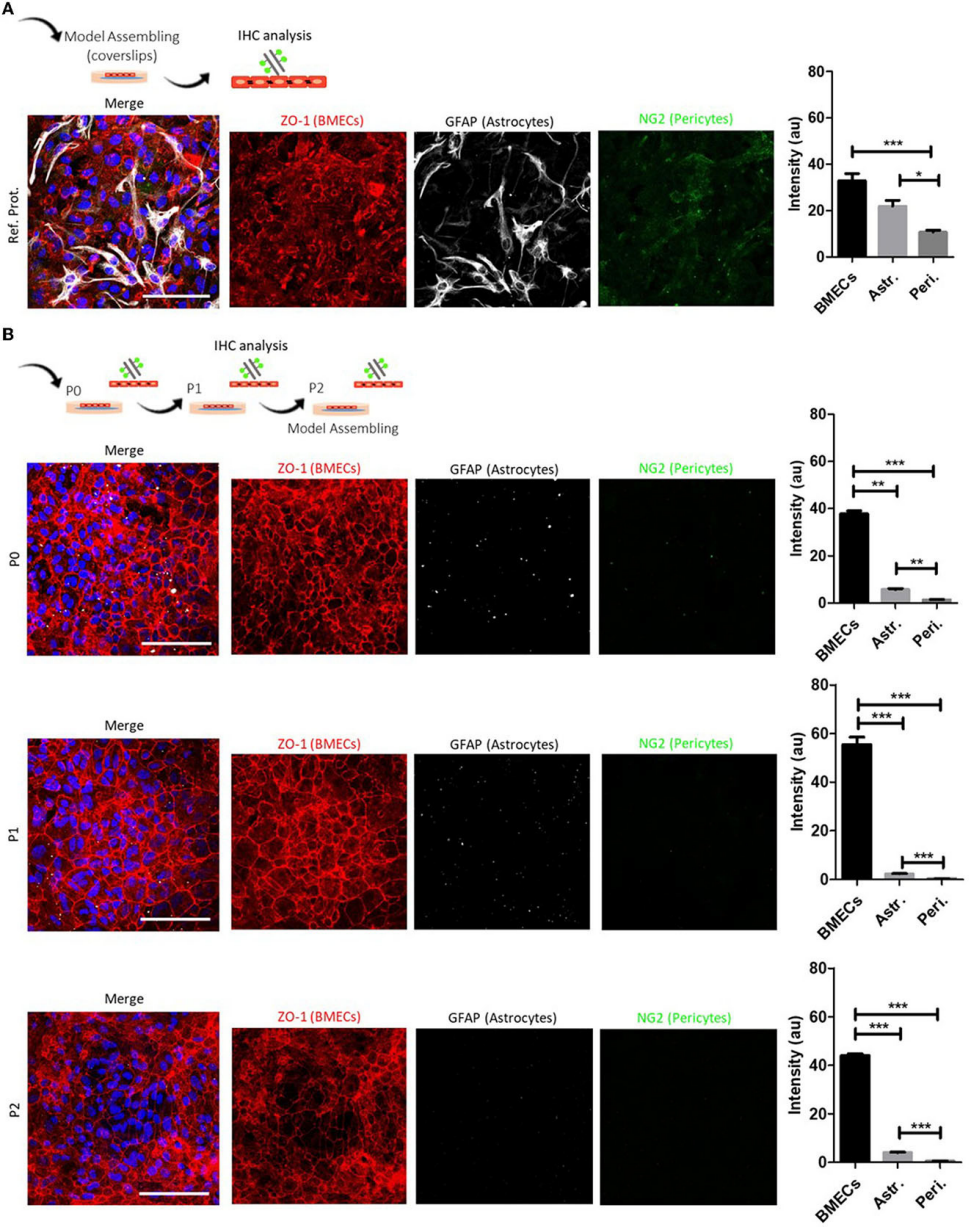
BMECs primary culture characterization and model assembling in coverslips. **(A)** Schematic representation of the methodology for the characterization of BMECs primary culture and representative composite confocal images of ZO-1/NG2/GFAP labeling. After reviewing BMECs protocols, we made a first primary culture adapted from our reference protocol (Xue et al., 2013). Quantitative analysis at P1 shows an abundant presence of BMECs (ZO-1) and astrocytes (GFAP). Pericytes (NG2) are found in less proportion. **(B)** Proposed BMECs primary culture protocol from newborn mice (PND1). Schematic representation of the methodology for the characterization of BMECs primary culture at P0, P1, and P2 and representative composite confocal images of ZO-1/NG2/GFAP labeling. Quantitative analysis during P0 to P2 shows an increased proportion of BMECs (ZO-1) when compared with astrocytes (GFAP) and pericytes (NG2). Nuclei are stained with DAPI (blue). *N* = 2. Scale bar 50 μm.

Accordingly, to develop this protocol, we first reviewed six published protocols for isolation and primary culture of BMECs obtained from mouse and rat brains (Nakagawa et al., 2009; Xue et al., 2013; Burkhart et al., 2015; Wang et al., 2015; Bernard-Patrzynski et al., 2019; information summarized in Table 5). We mainly focused on common elements and procedures, intending to integrate and provide relevant modifications presented in the step-by-step protocol. Based on the published protocol by Xue et al. (2013), we performed initial isolation of BMECs, followed by the primary culture that showed abundant growth of astrocytes and pericytes (Figure 2A; Supplementary Figure 1). Aiming to use newborn mice and reduce astrocyte and pericyte contamination, we developed the protocol presented here (Figure 2B; Supplementary Figure 2). We performed six separate isolation and BMECs primary culture rounds for validation purposes. During isolation and dissociation, we used two tissue digestion steps (trypsin and collagenase), the addition of BSA for myelin sheath separation, and reduced the number of centrifugation steps. We avoided Percoll density gradient cell separation, a procedure commonly used for the isolation and purification of BMECs (Table 5) (Nakagawa et al., 2009; Burkhart et al., 2015; Bernard-Patrzynski et al., 2019). We found that the cells were adequately disaggregated from the tissue with these steps sequence, recovering approximately 4.2 × 10^6^ (± 1.5 × 10^6^) viable cells (Table 6). In addition, most protocols use puromycin to avoid astrocytes and pericyte growth. Puromycin is one of the most used antibiotics, which inhibits protein synthesis by disrupting peptide transfer on ribosomes, causing premature chain termination during translation, and rapid cell death, in both prokaryotic and eukaryotic cells. In the case of BMECs primary culture, puromycin is widely used for cell culture purification, avoiding the growth of contaminant cell populations. Puromycin may act as a substrate in producing P-glycoprotein 1 (P-gp), a membrane protein highly expressed in BMECs but not in pericytes and glial cells. P-gp is an ATP-dependent efflux pump involved in defense mechanisms against harmful substrates. Specifically, endothelial cells of the BBB pump toxins out of the cell into the capillaries (Jetté et al., 1993). Thus, BMECs survive puromycin treatment without producing any effect on viability and phenotype (Jetté et al., 1993; Schumacher and Mollgård, 1997; Perriere et al., 2005). However, long-period treatments may produce cytotoxic effects (Perriere et al., 2005). In the same line, another work in drug testing triage, evaluated puromycin treatment in primary cell culture of BMECs from 30 days old C57BL6J mice and the mouse endothelioma cell lineage Bend.3. The authors tested several puromycin concentrations (0.01 to 100 μg/ml) and described that Bend.3 cells presented reduced cell viability after puromycin treatment (40–50%). Meanwhile, BMECs exhibited high cell viability (80–100%) independent of the puromycin treatment concentration (Puscas et al., 2019). In our experience, the use of puromycin caused detrimental effects on the viability of BMEC primary culture (P0) from newborn mice. We tested different concentrations of puromycin, from 0.5, 1.0, and 2.0 μg/ml for 48 h, 1 day after isolation and seeding (P0), and all treatments resulted in cell death (Supplementary Figure 5). Puromycin treatments in the following passages were not tested. Thus, according to our results, BMECs from neonatal mice (P0) failed to survive the puromycin treatment probably due to the immature phenotype of the cells.

In the same line, the regulated transport of some compounds across the BBB is essential in supporting brain development and function (Pardridge, 2012). The angiogenesis of fetal BMECs in humans may be related to P-gp (Barakat et al., 2008). *In vitro* studies on human primary fetal BMECs detected P-gp in capillary-like microvessels, cytoplasm, and cell nuclei through immunolocalization (Lye et al., 2022). Tsai et al. (2002) showed that in the mouse brain, P-gp expression is lower both during late embryogenesis and early neonatal period and increases gradually during brain maturation (P21), where P-gp protein levels approached adult levels. Nevertheless, even though P-gp protein and ABCB1 gene expression are lower during early and mid-gestational periods, its function does not change, and it works enough to protect BBB (Lye et al., 2022). The lower expression seems to be conserved since it was observed in mice, rats, and humans (Schinkel et al., 1994, 1995; Matsuoka et al., 1999; Watchko et al., 2001; Tsai et al., 2002; Daood et al., 2008). Although, there is a disconnection between P-gp functionality and its protein and gene expression in BMECs (Eustaquio Do Imperio et al., 2021). Therefore, exposure to xenobiotics, stress, and inflammatory stimuli can disrupt BBB permeability from low P-gp expression in fetal and early postnatal life (Bendayan et al., 2002; Goralski et al., 2006; Eng et al., 2022). Given these circumstances, we avoided puromycin treatment and only used cell medium enrichment with sequential passaging (5–6 div in P1 and P2, 80–85% confluence). The basic Fibroblast Growth Factor (bFGF) is a constitutive protein in BMECs (Schechter et al., 1996), regularly used as an *in vitro* media supplement intended to stimulate proliferation (Goto et al., 1993; Schechter et al., 1996; Caldwell et al., 2004; Yue et al., 2006). We supplemented the cell culture medium with 1.0 ng/ml bFGF after cell isolation and seeding (P0) and 20 ng/ml for P1 and P2. We found that bFGF supplementation combined with sequential passaging promoted a homogeneous population of BMECs (ZO-1) (Figure 2B).

**Figure 3:**
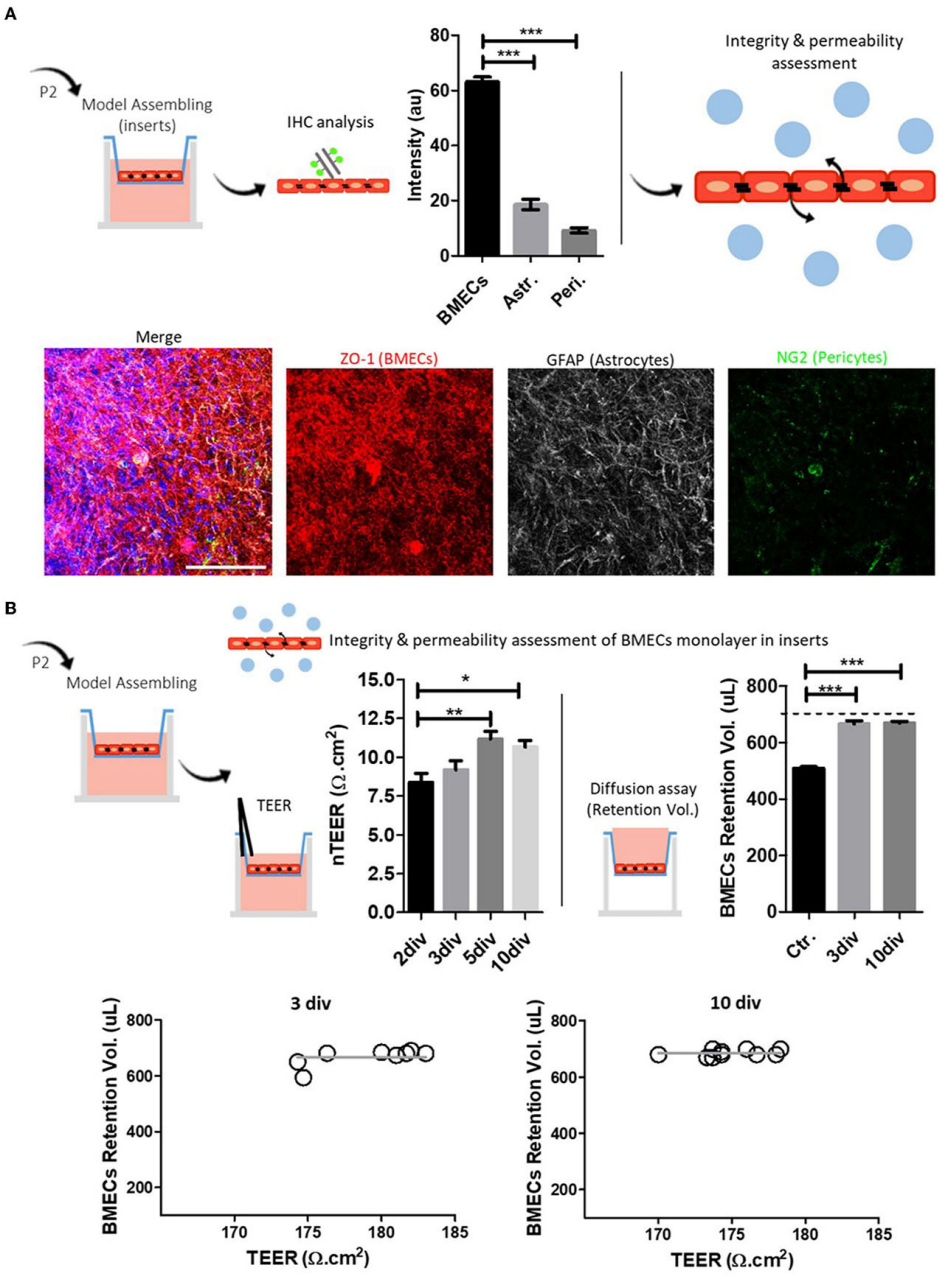
BMECs primary culture and model assembling in inserts. **(A)** Schematic representation of the methodology for the characterization of BMECs primary culture and representative composite confocal images of ZO-1/NG2/GFAP labeling; Quantitative analysis shows an increased proportion of BMECs (ZO-1). Astrocytes (GFAP) and pericytes (NG2) are also recognized. **(B)** Integrity and permeability assessment of the BMECs monolayer in inserts. Schematic representation of TEER and diffusion assay. TEER results gradually increased, reaching the highest value at 5 div. The diffusion assay showed similar results for 3 and 10 div. TEER and diffusion assay relationship show that TEER (3 and 10 div) values, either at 3 or 10 div, remained independent from the basal diffusion assay. One-way ANOVA and Bonferroni's multiple comparison test (* *p* < 0.05, ** *p* < 0.01, *** *p* < 0.001). Nuclei are stained with DAPI (blue), *N* = 3, Scale bar 50 μm.

We performed six separate procedure replicates of isolation and BMEC primary culture. We used light microscopy assessment during seeding and growth to follow the progression of the BMEC primary culture. During the first 24 h (1 div), adhesion of cells to the surface of the culture substrate was observed, as well as a high number of dead cells in suspension. During the following days (4–5 div), we observed a characteristic polygonal to spindle-shaped morphology and tightly packed monolayers. On day 7 of P0 (7 div), cell culture showed a confluence of around 90–95%, and a confluent cell monolayer was observed (Figure 1G). At this point, we recommend cell passaging (P1 and P2) with no more than 5–6 div between each passage (confluence 80–85%), allowing the purification and enrichment of BMECs pool while other contaminant cells, such as astrocytes and pericytes, perished. Image analysis of BMEC primary cultures at P0, P1, and P2 in coverslips showed an increase in the endothelial cell marker and tight junction protein ZO-1. Moreover, we found scarce labeling of GFAP, a protein expressed by astrocytes and neural progenitors (Escartin et al., 2021), and NG2, a transmembrane proteoglycan expressed by pericytes (Brand et al., 2016; Naranjo et al., 2021) (Figure 2). We also found a similar pattern of labeling and distribution (intensities) for BMECs cultured on inserts. BMECs effectively formed a tightly connected cell barrier on the surface of the insert between 3 and 5 div. However, at 10 div, we observed enrichment of astrocytes and pericytes (Figure 3A). These immunocytochemical results are in accordance with the compared protocol for primary endothelial cell isolation and culture (Perriere et al., 2005; Wisniewska-Kruk et al., 2012; Xue et al., 2013). Finally, we also evaluated the integrity and permeability of BMECs monolayer through the association of a flux/diffusion assay and TEER measurement, both recommended methods for the optimal characterization of the paracellular permeability (Helms et al., 2016). In BMECs seeded in transwell-insert systems, TEER is a general measure of the paracellular flux of ions and molecules between cells, which allows the evaluation of cell barrier function (Hoheisel et al., 1998). Thus, for this work, we performed a cell medium diffusion test in control BMECs systems (adapted from Wang et al., 2015) and a FITC-DEXTRAN diffusion assay in BMECs submitted to a model of hypoxia (OGD).

First, we performed TEER measurements in primary human umbilical cells (HUVEC) monolayer inserts at 2 and 3 div to validate the methodology (Supplementary Figure 3), repeated at least three times. HUVEC-TEER measurements at 2 and 3 div follow previously published data (Tschugguel et al., 1995; Dewi et al., 2004; Cucullo et al., 2008), showing comparable TEER values (Dewi et al., 2004) and the differential response to hypoxic conditions (Pairet et al., 2019). We next performed TEER measurements on BMECs monolayers at 2, 3, 5, and 10 div. Results showed an increased resistance at 3, 5, and 10 div compared to 2 div (Figure 3B). Similar TEER patterns were previously seen in related paper protocols (Eigenmann et al., 2013; Patabendige et al., 2013). In parallel, the cell medium diffusion test showed that BMECs monolayer cultured on inserts for 3 and 10 days effectively retained the medium in the apical side of the insert compared to control, cell-free inserts (Figure 3B).

Regarding the suitability of this BMECs culture protocol and the possible biological explanation of these results, we mainly suggest that long-period cultures in transwell-inserts (which, in our case, refers to more than 5 div) may potentially compromise the integrity and permeability of the cell culture system. In this context, we found at least two explanations: (i) the progressive outgrowth and enrichment of contaminant cell lineages—astrocytes and pericytes—overtime affecting the integrity of the BMECs monolayers and, in consequence, TEER values in the long-term and (ii) the initial seeding density and other associated rate of cell growth. For the first assumption, we found controversial data. While a previous study showed that puromycin-purified rat BMECs culture exhibited improved barrier properties, including TEER values (Calabria et al., 2006), several works also demonstrated that BMECs co-cultured with pericytes and astrocytes might enhance the conditions of the primary culture system (Abbott, 2002; Siddharthan et al., 2007; Wisniewska-Kruk et al., 2012; Jamieson et al., 2019; Rado et al., 2022). On the other hand, cell culture seeding density impact the viability and suitability of the cell culture over time. Papers reviewed for the isolation and primary culture of BMECs mentioned a cell density seeding between 1.5 × 10^5^ and 1.0 × 10^7^. Our protocol used 2.0 × 10^5^ cells for polycarbonate insert transwell (0.4 μm pore, 0.47 cm^2^ surface area), a quantity that is within the quoted values. Previous work focused on optimizing BMEC primary cultures, which evaluated different seeding densities, showed that there may be a minimum number of cells required to obtain a tight barrier composed of endothelial cells (Wuest and Lee, 2012). The work also showed that larger densities cultures (up to 8 × 10^5^ cells/cm^2^) reached their maximum TEER value faster than low seeding densities (1 × 10^5^ and 2 × 10^5^ cells/cm^2^). However, after their maximum TEER value, BMECs showed drastically decreased values, a condition not observed with other seeding densities. The authors suggested that decreased TEER values may be attributed to a loosening of the monolayer, likely due to cells dying and losing the ability to form intercellular tight junctions. The work concluded that the more favorable seeding density might be 4 × 10^5^ cells/cm^2^ (Wuest and Lee, 2012).

Finally, we analyzed the relationship between TEER values and flux/diffusion assay (Figure 3B). Our results suggest that TEER values, either at 3 or 10 div, remained independent from the basal diffusion assay. Similar results were also found in previous works from other authors (Gaillard and de Boer, 2000; Patabendige et al., 2013). Specifically, Gaillard and de Boer (2000) using two paracellular permeability assays, sodium fluorescein (FLU) and 4 kDa FITC-DEXTRAN (FDA4), showed an apparently (non-linear) relationship between basal permeability (FLU and FDA4) and TEER values, which was described by the one-phase exponential decay model. This non-linearity relationship can be explained by the fact that solute transport in FLU and FDA4 assays essentially depends on the sum of transport across a junction pathway. On the other hand, total electrical resistance, obtained by TEER measurements, is essentially sensible to areas with the lowest electrical resistance between single cells, even when these areas are present at a low density.

### BMECs response to the OGD model of hypoxia

**Figure 4:**
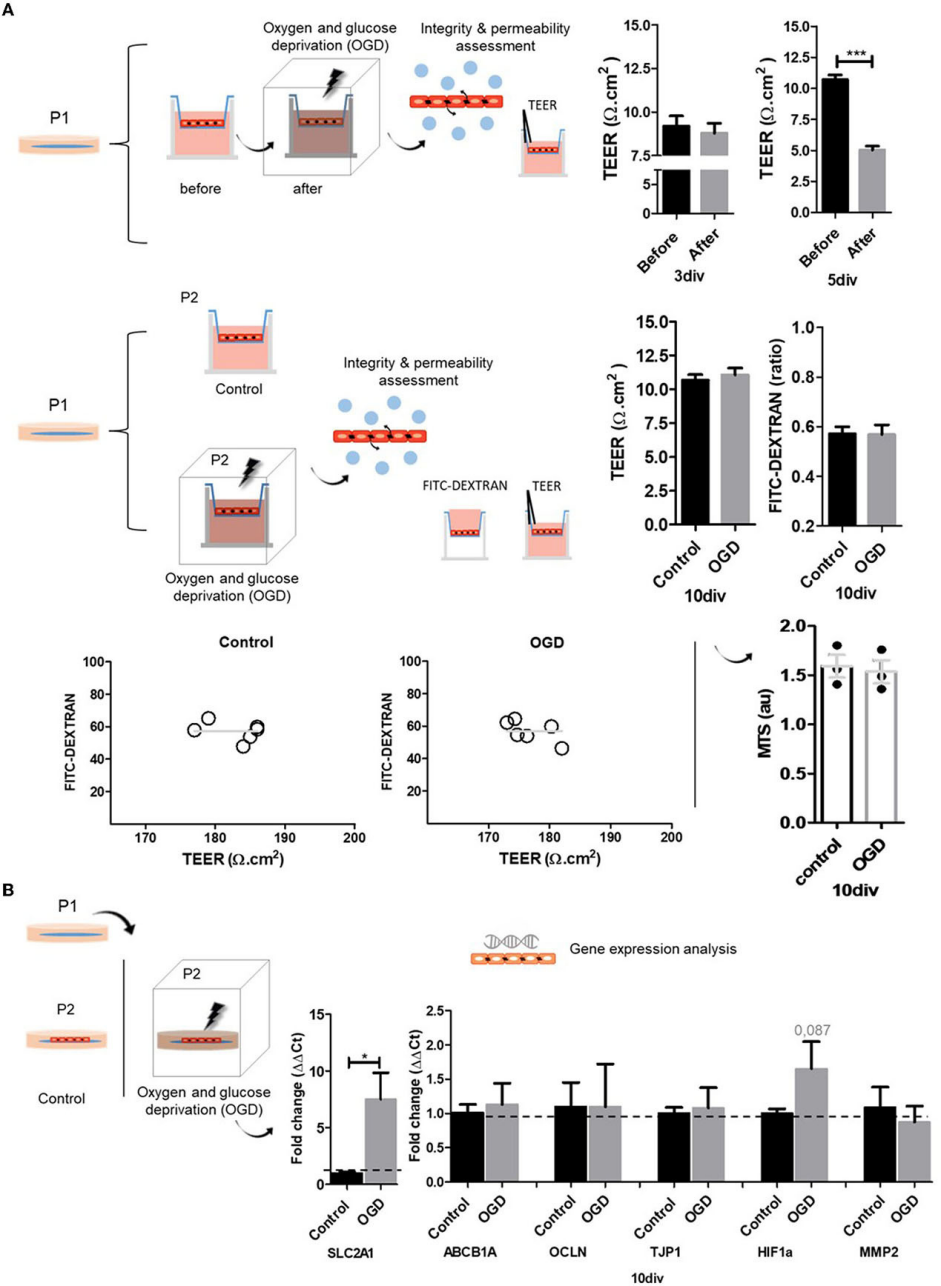
Oxygen and glucose deprivation (OGD) model of hypoxia. **(A)** We use two OGD experimental designs. a before-after and a control-OGD samples. We measured TEER, diffusion/FITC-DEXTRAN, and MTS. In the case of the before-after approach, at 3 div, OGD-BMECs primary cultures in inserts showed a slight reduction in TEER results, with no significant differences. At 5 div, OGD-BMECs primary cultures in inserts showed significant differences in TEER results. Control-OGD experimental design does not show changes at 10 div. TEER and diffusion assay relationship showed that TEER values remained independent from the FITC-DEXTRAN results. Cell viability (MTS) analysis at 10 div does not show differences between control and OGD. **(B)** OGD experimental design (control and OGD) and gene expression analysis (qPCR). At 10 div, there was an increased expression of the SLC2A1 gene after OGD. ABCB1A, OCLN, TJP1, HIF1a, and MMP2 genes do not show significant changes. Nuclei are stained with DAPI (blue). *N* = 3. T-Test (* *p* ≤ 0.05, *** *p* ≤ 0.001).

Finally, we tested BMECs function in a model of oxygen-glucose deprivation (OGD). BMECs dysfunction, and BBB disruption, are major features of acute neurological conditions such as stroke. In this line, stroke leads to ischemia and inflammation, promoting tissue reduced oxygenation or hypoxia (Jiang et al., 2018; Kunze and Marti, 2019; Yang et al., 2019; Archie et al., 2021; Mitroshina et al., 2021). Given this scenario, we subjected both BMECs models, glass coverslips and, inserts, to a 4-h OGD condition, an *in vitro* model of hypoxia. We mainly focused on evaluating the integrity and permeability of BMECs monolayer using TEER and FITC-DEXTRAN assay. For TEER measurements were considered two experimental designs: a paired measurement, before and after OGD, and as independent measurements, control, and OGD. Before-after OGD TEER measurements were recorded in BMECs cultures with two seeding periods, 3 and 5 div. Independent control and OGD samples were evaluated at 10 div. Additionally, we conducted gene expression profiles of related BMECs and hypoxia response genes in BMECs cultures at 10 div. OGD promoted a slightly reduced integrity and permeability (TEER) of BMECs cultures at 3 div. However, cultures at 5 div showed a significant decrease (Figure 4A). On the other hand, analysis of control and OGD samples did not show significant differences (10 div) in TEER and FITC-DEXTRAN assays and showed similar TEER and FITC-DEXTRAN relations. These results suggest that a paired experimental design for TEER measurements may be the best for identifying causal relationships. Nevertheless, we measured cell viability by MTS, given that our experimental design included a 4-h OGD treatment which might create rapid cell loss by necrosis. Our analysis did not show statistically significant differences. However, we noted a slight decrease in OGD samples. MTS is a colorimetric method for determining the number of viable cells, commonly used in proliferation, cytotoxicity, or chemosensitivity assays, where absorbance is directly proportional to the number of living cells. Although it is not the focus of this work and given this slight difference between control and OGD samples, we recommend the association of cell viability with cytotoxicity assays as an indicator of whole metabolic activity, avoiding misleading results. Commonly used assays for cell viability measure ATP levels, protease activity, and mitochondrial metabolic activity. On the other hand, cytotoxic assays detect the appearance of certain proteins after cell death, such as enzymes (protease and lactate dehydrogenase), or DNA-binding dyes which enter and stain permeable dead cells. Finally, we analyzed the expression profile of genes related to tight junctions (tight junction protein 1 TJP1 and occludin), efflux and glucose transporter (SLC2A1 and ABCB1a), and angiogenesis-hypoxia response (HIF-1α, MMP-2) in control and OGD BMECs primary cultures (Figure 4B). SLC2A1 and ABCB1a are solute carrier/transporters. SLC2A1, also known as GLUT-1, is a BMEC protein fundamental for capillary formation (Goldeman et al., 2020; Veys et al., 2020; Garcia et al., 2022), and the HIF-1α signaling target. During hypoxia, stabilization of HIF-1α and activation of the hypoxia signaling cascade, SLC2A1 expression increases to allow cell glucose absorption. Our analysis shows the upregulation (~seven-fold change) of SLC2A1 after OGD (Figure 4). ABCB1a gene encodes P glycoprotein, which is ubiquitously expressed in BMECs (Bendayan et al., 2002). Our study did not show differences between control and OGD, a result that was also found in a previous study with hCMEC/D3 cell lineage (Patak et al., 2011). The study suggested that the regulation of ABCB1 and ABCC1 may depend on different factors in addition to hypoxia, such as glucose deprivation and reoxygenation. In parallel, other work showed upregulation of ABCB1 gene expression after hypoxia (Lindner et al., 2012). OCLN and TJP1 genes are fundamental for BMEC's tight junction formation. The Tight Junction Protein 1 gene (TJP1) encodes ZO1, an adherent junction adaptor, a member of the membrane-associated guanylate kinase family. Tight junctions regulate the movement of ions and macromolecules between endothelial and epithelial cells. A recent single-cell characterization study of the human cerebral vasculature showed downregulation of CLDN5 and TJP1, which leads to the loss of BBB integrity in Huntington's disease (Garcia et al., 2022). Although HIF-1α expression was higher after OGD, the increase was not statistically significant (*p* = 0.087; Figure 4). In normoxia, HIF-1α is constitutively expressed and degraded, and under hypoxic conditions, HIF-1α is stabilized and activates the hypoxia signaling cascade (Ogunshola and Al-Ahmad, 2012; Engelhardt and Liebner, 2014). HIF-1α is expressed by different cell types and has a half-life of fewer than 5 min in normoxia (Shi, 2009; Semenza, 2014; Sun et al., 2017). The HIF-1α pathway modulates the expression of more than 200 genes, being an important regulator of the expression of vascular growth factor (VEGF) and matrix metalloproteases (MMP-2, MMP-9) that participate in the BBB disruption process (Turner and Sharp, 2016; Zhang et al., 2018; Page et al., 2019). MMP2 gene encodes the enzyme gelatinase A, a type IV collagenase of the matrix metalloproteinase (MMP) family found in the extracellular matrix and membrane of endothelial cells, actively secreted by endothelial cells during angiogenesis and neurological conditions (Arkell and Jackson, 2003). In our assay, we did not find differences in the expression of MMP2 between control and OGD cultures.

During the last decade, several models of BBB *in vitro* based on two-dimensional (2D) cell culture have been proposed. Using these models has contributed to the knowledge of BBB's physiology, pathology, and pharmacology. *In vitro* characterization and validation of the primary culture of BMECs monolayers seeded in glass coverslips and inserts include the assessment of the cells by light microscopy during culture, immunolabeling, and gene expression profile. In addition, transendothelial electrical resistance (TEER) measurement was used as a functional assay of the adherent junction complexes. Altogether, the protocol presented here for the isolation and culture of BMECs is more straightforward than previously published protocols, yields a high number of purified cells, and may be suitable for use as a bioscaffold for secondary cell seeding allowing the study and better understanding of the NVU.

## Conclusion

Our protocol for obtaining BMECs represents a high throughput isolation method without requiring additional substances for cell separation and purification. Other sources of BMECs to establish BBB models, such as immortalized cells or pluripotent stem cell-derived, affect TEER values and demand high-cost maintenance, respectively, our BMECs primary culture possess the advantage of preserving BBB characteristic properties and has a lower maintenance cost. Altogether, we present a protocol to effectively isolate and grow BMECs from newborn mice, and provide evidence for the consistency of this protocol and the properties of the primary culture.

## Data availability statement

The raw data supporting the conclusions of this article will be made available by the authors, without undue reservation.

## Ethics statement

The animal study was reviewed and approved by Ethics Committee on the Use of Animals of the Universidade Federal de São Paulo (CEUA/1344290719).

## Author contributions

PN-A performed the experiments and the analyses, wrote the original draft, and reviewed and edited the document table and figures. LD-G, TN, AM, and NC performed experiments and analyses, contributed the original draft, and reviewed and edited the document table and figures. MP conceptualized the study, advised in the execution of the experiments and analyses, and reviewed and edited the final document. All authors contributed to the article and approved the submitted version.

## Funding

This work was supported by the São Paulo Research Foundation (FAPESP) (Grant Numbers 2018/12605-8 and 2016/19084-8), Coordination for the Improvement of Higher Education Personnel (CAPES; Financial Code 001), and the National Council for Scientific and Technological Development (CNPq) (Grant Numbers 465656/2014-5, 309679/2018-4, 141907/2018-5).

## Conflict of interest

The authors declare that the research was conducted in the absence of any commercial or financial relationships that could be construed as a potential conflict of interest.

## Publisher's note

All claims expressed in this article are solely those of the authors and do not necessarily represent those of their affiliated organizations, or those of the publisher, the editors and the reviewers. Any product that may be evaluated in this article, or claim that may be made by its manufacturer, is not guaranteed or endorsed by the publisher.

## Footnotes

^1^Stroke Progress Review Group. https://www.ninds.nih.gov/stroke-progress-review-group.

^2^ https://www.ninds.nih.gov/About-NINDS/Strategic-Plans-Evaluations/Strategic-Plans/Stroke-Progress-Review-Group

^3^ http://rsbweb.nih.gov/ij

## Supplementary material

Supplementary Figure 1 Primary culture adapted from the reference protocol (Xue et al., 2013). Schematic representation of the methodology for the characterization of BMECs primary culture and representative composite confocal images of ZO-1/NG2/GFAP labeling.

Supplementary Figure 2 BMECs primary culture from newborn mice. Schematic representation of the methodology for the characterization of BMECs primary culture at P0, P1, and P2, and representative composite confocal images of ZO-1/NG2/GFAP labeling.

Supplementary Figure 3 TEER validation methodology. HUVEC samples were used to validate the TEER methodology. Results remain in correspondence with previously published works. At 3 div, there is an increase in TEER measurement. In parallel, after OGD, TEER measurements significative decreased. N=6 Test (^**^ *p* ≤ 0.01, ^***^ *p* ≤ 0.001).

Supplementary Figure 4 TEER electrode's support. Top. Representative images of TEER electrode's support and set up of the experiment. Bottom. Technical information and design of the support.

Supplementary Figure 5 BMECs primary culture and puromycin treatment. Representative light microscopy images of, control (without treatment), and puromycin treatments: 0.05, 1 and 2 μg/ml. Puromycin treatment caused cell death of BMECs primary culture.
