# Supplementary figures and images for: Simple and efficient protocol to isolate and culture brain microvascular endothelial cells from newborn mice
Source: Front Cell Neurosci. 2022 Oct 13;16:949412. doi: 10.3389/fncel.2022.949412 (PMC9606660; doi:10.3389/fncel.2022.949412)

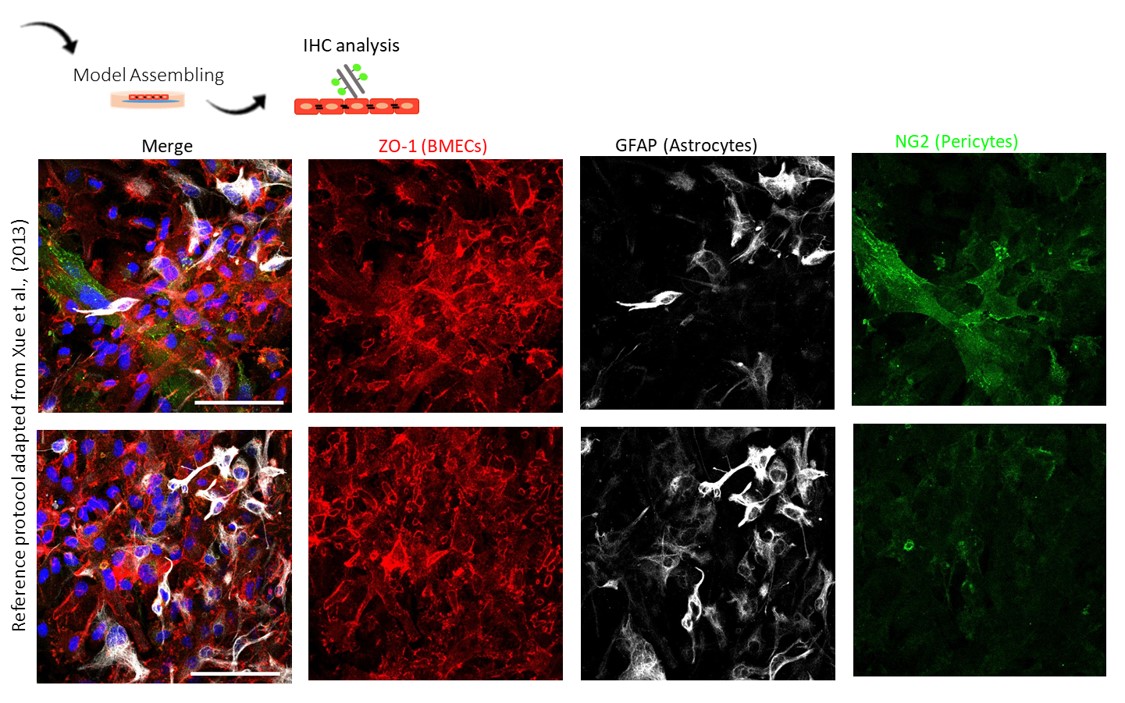

Supplement: Supplementary Figure 1 — Primary culture adapted from the reference protocol (Xue et al., 2013). Schematic representation of the methodology for the characterization of BMECs primary culture and representative composite confocal images of ZO-1/NG2/GFAP labeling. [file Image_1.jpg]

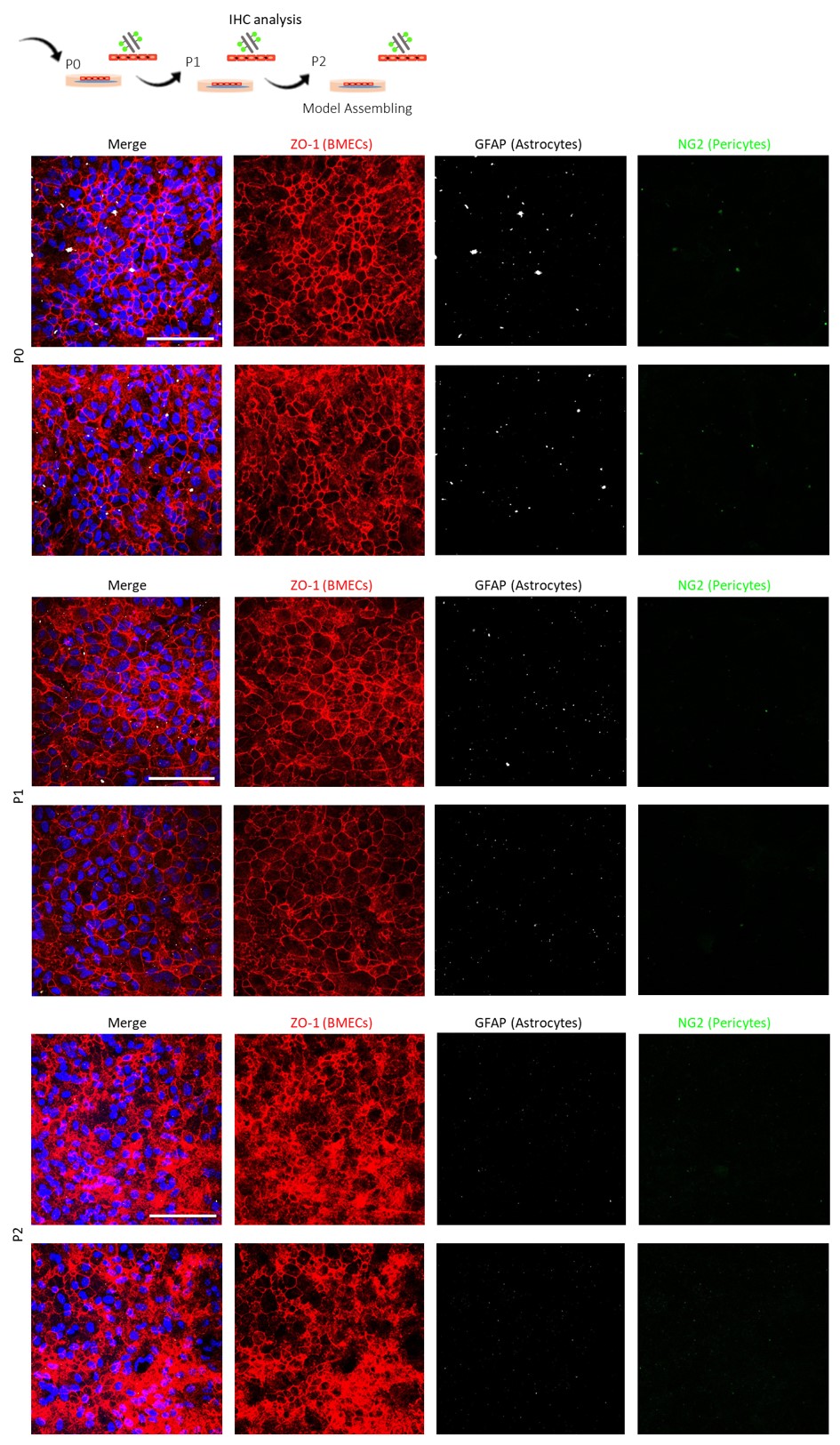

Supplement: Supplementary Figure 2 — BMECs primary culture from newborn mice. Schematic representation of the methodology for the characterization of BMECs primary culture at P0, P1, and P2, and representative composite confocal images of ZO-1/NG2/GFAP labeling. [file Image_2.jpg]

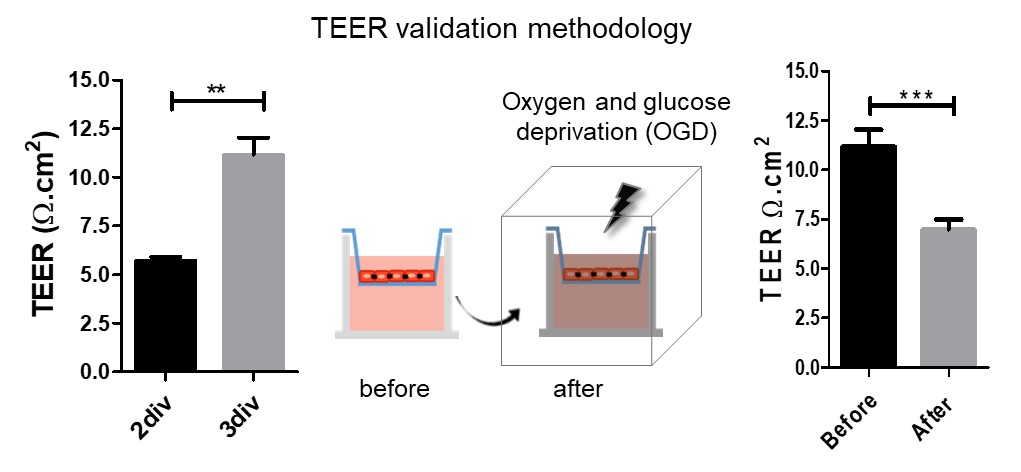

Supplement: Supplementary Figure 3 — TEER validation methodology. HUVEC samples were used to validate the TEER methodology. Results remain in correspondence with previously published works. At 3 div, there is an increase in TEER measurement. In parallel, after OGD, TEER measurements significative decreased. N=6 Test (** p ≤ 0.01, *** p ≤ 0.001). [file Image_3.jpg]

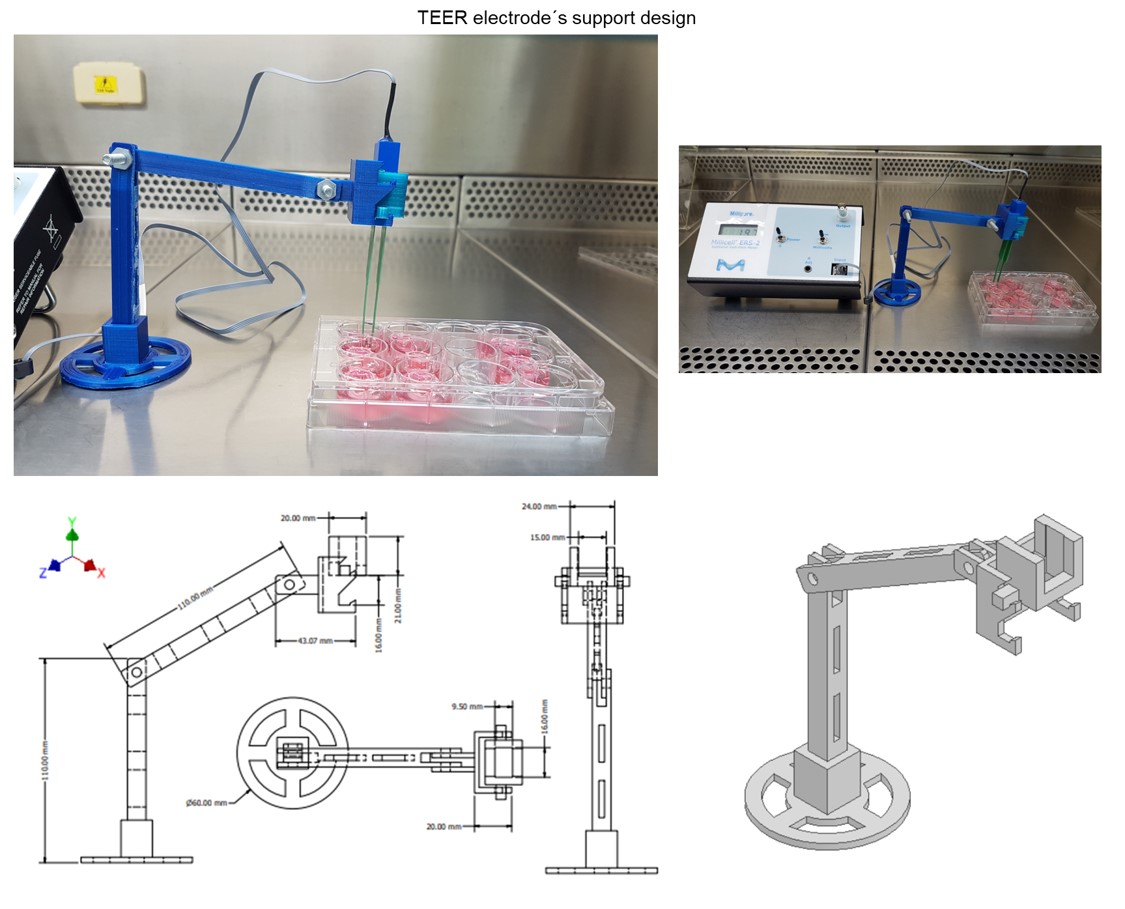

Supplement: Supplementary Figure 4 — TEER electrode's support. Top. Representative images of TEER electrode's support and set up of the experiment. Bottom. Technical information and design of the support. [file Image_4.jpg]

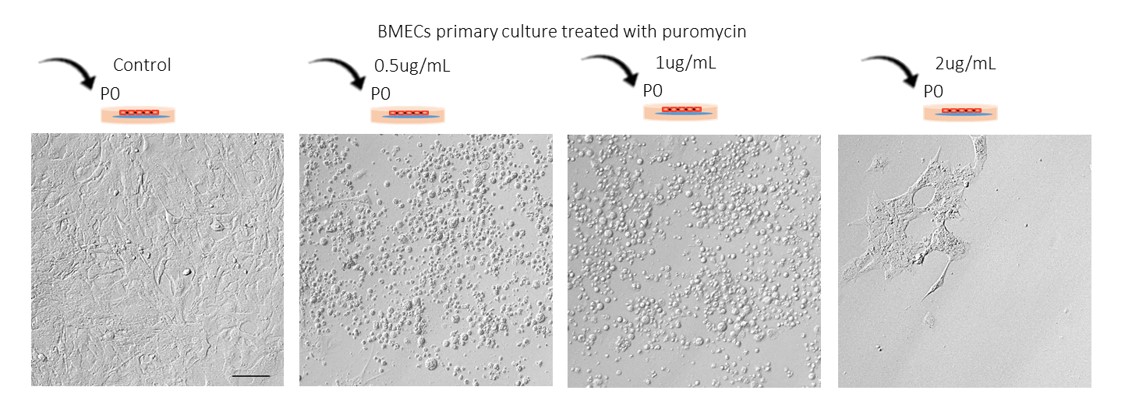

Supplement: Supplementary Figure 5 — BMECs primary culture and puromycin treatment. Representative light microscopy images of, control (without treatment), and puromycin treatments: 0.05, 1 and 2 μg/ml. Puromycin treatment caused cell death of BMECs primary culture. [file Image_5.jpg]
